# Transcranial Direct Current Stimulation to Enhance Dual-Task Gait Training in Parkinson’s Disease: A Pilot RCT

**DOI:** 10.1371/journal.pone.0158497

**Published:** 2016-06-30

**Authors:** Siobhan M. Schabrun, Robyn M. Lamont, Sandra G. Brauer

**Affiliations:** 1 Brain Rehabilitation and Neuroplasticity unit, School of Science and Health, Western Sydney University, Penrith, NSW, Australia; 2 School of Health and Rehabilitation Sciences, The University of Queensland, St Lucia, Brisbane, Queensland, Australia; St Francis Hospital, UNITED STATES

## Abstract

**Objective:**

To investigate the feasibility and safety of a combined anodal transcranial direct current stimulation (tDCS) and dual task gait training intervention in people with Parkinson’s Disease (PD) and to provide data to support a sample size calculation for a fully powered trial should trends of effectiveness be present.

**Design:**

A pilot, randomized, double-blind, sham-controlled parallel group trial with 12 week follow-up.

**Setting:**

A university physiotherapy department.

**Interventions:**

Sixteen participants diagnosed with PD received nine dual task gait training sessions over 3 weeks. Participants were randomized to receive either active or sham tDCS applied for the first 20 minutes of each session.

**Main Measures:**

The primary outcome was gait speed while undertaking concurrent cognitive tasks (word lists, counting, conversation). Secondary measures included step length, cadence, Timed Up and Go, bradykinesia and motor speed.

**Results:**

Gait speed, step length and cadence improved in both groups, under all dual task conditions. This effect was maintained at follow-up. There was no difference between the active and sham tDCS groups. Time taken to perform the TUG_words_ also improved, with no difference between groups. The active tDCS group did however increase their correct cognitive response rate during the TUG_words_ and TUG_count_. Bradykinesia improved after training in both groups.

**Conclusion:**

Three weeks of dual task gait training resulted in improved gait under dual task conditions, and bradykinesia, immediately following training and at 12 weeks follow-up. The only parameter enhanced by tDCS was the number of correct responses while performing the dual task TUG. tDCS applied to M1 may not be an effective adjunct to dual task gait training in PD.

**Trial Registration:**

Australia-New Zealand Clinical Trials Registry ACTRN12613001093774

## Introduction

Parkinson’s disease (PD) is a progressive neurological disorder characterized by deficits in gait and postural control. People with PD frequently walk with reduced gait speed and step length [1], and increased stride to stride variability [2]. When asked to perform a concurrent task while walking, for example thinking or holding an object, gait can deteriorate further[1, 3–6]. This has led to a recommendation that individuals with PD avoid dual tasking. However, independent living requires the ability to dual task, and recent evidence suggests that with training, individuals with PD can improve their ability to walk while dual tasking[7, 8]. For example, a 20 minute session of dual task walking training resulted in increased step length and gait speed[7]. Similarly, training using visual cues has been shown to improve stride length while dual tasking in PD[3]. Non-invasive brain stimulation has the potential to enhance dual task gait training and may result in larger, sustained improvements in gait performance in individuals with PD than can be achieved with training alone.

Successful dual tasking when walking requires both motor and cognitive involvement. For instance, a recent study reported altered functional connectivity in dual-tasking related brain networks such as the cerebellum and motor cortical areas in people with PD compared to healthy controls [9]. This finding was interpreted to reflect a compensatory strategy for reduced motor output stemming from striato-thalamo-cortical dysfunction. These data suggest that interventions that can increase motor activity may be beneficial for dual tasking in people with PD. Transcranial direct current stimulation (tDCS) can increase the activity of the ventro-posterolateral thalamic nucleus[10], suggesting effects on a distributed cortical network that may influence basal ganglia function[11]. In addition, anodal tDCS over the primary motor cortex (M1) is known to alter resting membrane potentials of underlying neurons leading to an increase in cortical excitability that has been proposed to help compensate for reduced pallido-thalamo-cortical drive[11–13]. Indeed, tDCS has been shown to improve gait, balance, motor function and bradykinesia when applied to the M1 of individuals with PD while at rest[11, 14, 15]. In addition, tDCS has been shown to boost the effect of physical gait training in PD, resulting in greater improvements in gait velocity when tDCS and physical training are combined than when either therapy is applied alone[14]. These improvements are reported after a single, 15-minute session of therapy and are more pronounced in individuals with greater motor impairment. These findings suggest that tDCS may be beneficial as a tool to improve dual tasking in people with PD.

To our knowledge, no study has investigated the effect of tDCS on dual task gait training in PD, despite promising effects when tDCS is combined with other forms of physical gait training. The aims of this pilot randomised controlled trial were to: i) determine the feasibility and safety of a combined tDCS and dual task gait training intervention in PD and ii) provide data to support a sample size calculation for a fully powered trial should trends for greater improvements in gait performance and motor function be present when anodal tDCS is combined with dual task gait training than when dual task gait training is applied alone.

## Materials and Methods

### Participants

**Fig 1:**
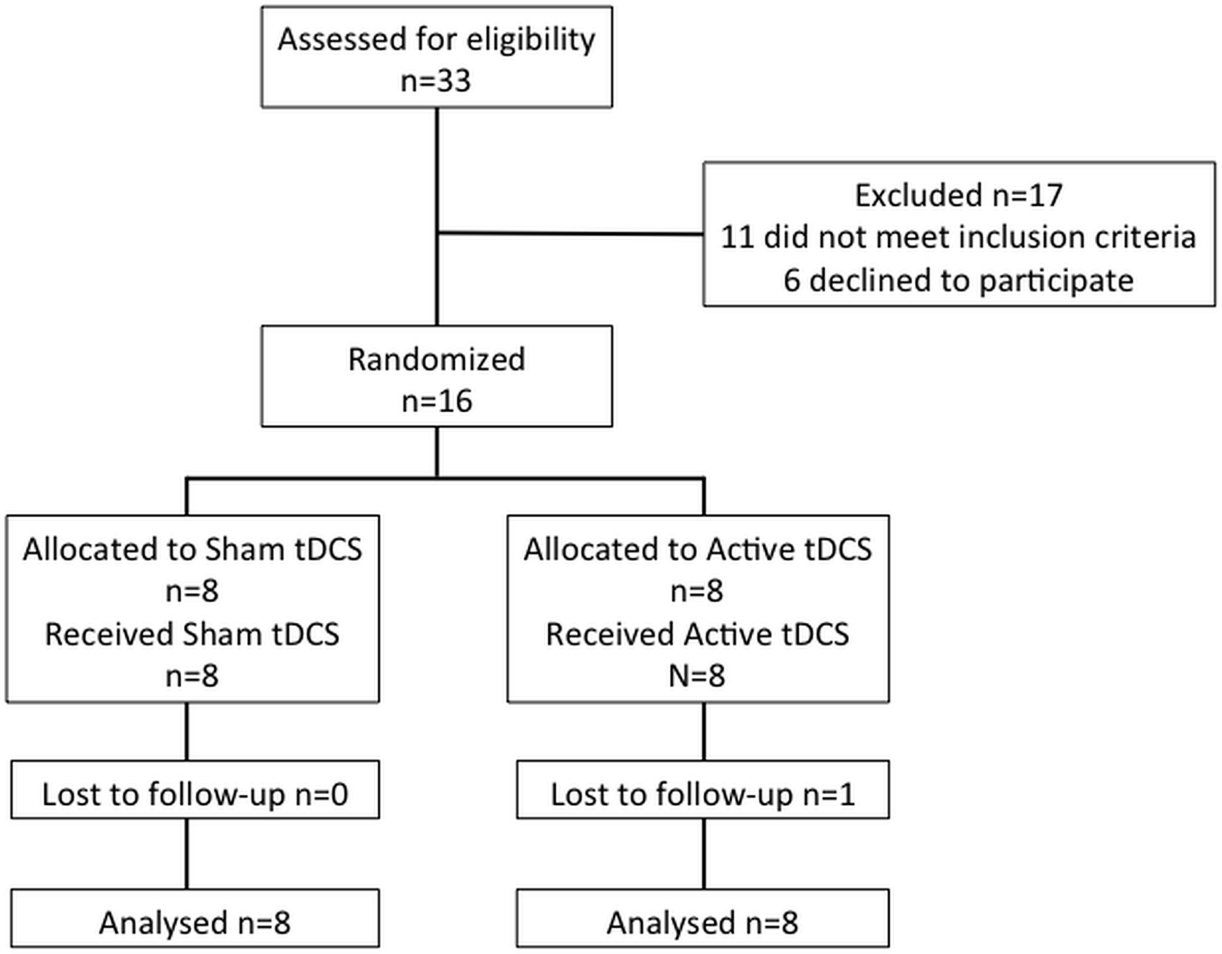
Flow diagram of participants through the trial.

As this was a pilot trial designed to generate data that can be used to inform a future large randomised controlled trial (should the intervention appear feasible, safe and show trends of effectiveness), a sample size of 24 participants was selected. This was based on previous studies that have shown a 5–20% change in gait speed after 8 sessions of tDCS alone with 13 participants per group[15] and an improvement of approximately 20% in gait speed with the physiotherapy dual task training alone[7]. However, due to slower than expected recruitment rates and strict exclusion criteria, a total of 33 participants diagnosed with idiopathic PD by a neurologist were screened for eligibility. Six declined participation and a further 11 did not meet the inclusion criteria (presence of co-morbidities such as stroke, deep brain stimulator, atypical PD or unclear diagnosis, fragile scalp, low cognition levels). Thus, sixteen participants were enrolled in the trial (Fig 1).

Participants were included if they were aged over 18 years, had mild-moderate disease severity (stage II-III on the Hoehn and Yahr scale), could walk 100m independently with or without gait aid, reported reduced step length or slowed gait speed confirmed by clinical examination, did not have metal objects or stimulators in the head that might pose a hazard during tDCS; had no known neurological conditions other than PD; had no known musculoskeletal or cardiopulmonary conditions that could affect the ability to walk safely; scored > 24 on the Mini-Mental Status Examination (MMSE)[16] and had no known sensory system pathology affecting walking or communication (e.g. blindness, deafness). This study was publicly registered (ACTRN12613001093774), approved by the University of Queensland’s Human Medical Research Ethics committee (S1 File) and complied with the declaration of Helsinki and the CONSORT statement (S2 File). All participants provided written, informed consent. The authors confirm that all ongoing and related trials for this intervention are registered.

### Trial design

A pilot, randomized, double-blind, sham-controlled parallel group trial with 12 week follow-up was undertaken. Outcome measures were assessed in the week pre and post training, and at 12 weeks follow up, in their self-reported optimally medicate state, or ‘ON’ period, typically one hour after medication. Participants were randomized into two groups; i) gait training and active tDCS or ii) gait training and sham tDCS. An offsite investigator not involved in recruitment, intervention or data collection prepared a concealed randomization schedule using a computer generated random number sequence. Consecutively numbered, randomly ordered opaque envelopes containing group allocation in a 1:1 ratio were opened after baseline assessment by the physiotherapist applying the tDCS. The physiotherapist conducting the gait training, the investigator conducting the assessments and the participants were blind to group allocation.

### Intervention

Training was provided in nine x 60-minute sessions across three weeks (three sessions per week) commencing at the patient’s self-reported optimal ‘ON’ period[7]. Active or sham tDCS was delivered during gait training by placing the tDCS device in a small bag positioned around the participant’s hips. All assessment and training occurred at a University physiotherapy department.

#### Transcranial direct current stimulation (tDCS)

Anodal tDCS was delivered using two saline soaked surface electrodes (35cm^2^) and a battery-operated unit (Magstim, UK). Current was applied with the anode positioned over the left primary motor cortex (M1) according to the 10/20 international system for EEG electrode placement and the cathode positioned over the contralateral supra-orbital region. [15] A constant current of 2 mA was applied during the initial 20 minutes of gait training[15]. For sham stimulation, electrodes were placed in an identical position to active stimulation but the current was ramped up over 10 seconds, down over 10 seconds and then switched off. This is a standard tDCS sham procedure that ensures participants feel the initial tingling sensation associated with tDCS[15]. The tDCS unit was placed out of sight in a small bag for both the active and sham interventions.

#### Gait training

Gait training was performed by a trained physiotherapist on a one-on-one basis using a protocol based on a previous trial[17]. Participants undertook repeated walking practice with visual, verbal or self-cueing aimed to improve step length and walking speed. Secondary cognitive and motor tasks were progressively integrated into the training program. Tasks were designed to reflect functional everyday activities and included listening, speaking, conversing, list recall and generation, calculation tasks, carrying bags, getting keys out of a pocket, counting money or recalling directions. Gait tasks were also made increasingly difficult by reducing or removing cues, adding obstacles and progressing from a simple, controlled walking environment to more natural and uncontrolled environments. Task complexity was progressed by increasing the difficulty of the gait or secondary task and by combining multiple tasks in one activity. In addition, participants were initially instructed to divide their attention equally between improving their gait and the secondary task. As able, participants were asked to vary the focus of their attention, attending more to their gait (80%) for a block of the training and more to the secondary task for a block of the training. The duration of each block was shortened over the three weeks so that participants were more frequently changing the focus of their attention. Using a visual analogue scaled from 0–100%, participants were intermittently asked to indicate how much of their attention was focused on their walking.

### Outcome measures

The primary outcome was gait velocity (m/s) when walking over 8m and undertaking one of three concurrent cognitive tasks (word lists, counting, conversation: Gait_words_, Gait_count_ and Gait_conversation_). Secondary gait parameters included cadence (steps/min), step length (m) and double support time (s). These parameters were chosen as they show performance decrements in people with PD during dual tasking. Gait parameters were assessed using a GAITRite**®** electronic walkway that has been shown to have excellent test-retest reliability in older adults, is sensitive to gait alterations in individuals with PD and is considered the gold standard comparator when investigating gait during dual tasking in people with PD[18, 19]. In the Gait_words_ and Gait_count_ tasks, participants walked at their comfortable pace across a 10m unobstructed path over the 8m GAITRite**®** mat, and were instructed to perform both the walking and added task to the best of their ability. In the conversation task, participants walked an indoor circuit for 3 minutes where they were engaged in conversation for 80% of that time. In the first and last 8m of the path participants walked over the GAITRite mat**®**. To assess whether dual-tasking improved independent of gait performance, the number of correct responses (numbers or words) was calculated during gait (time taken to walk 8 m) for the Gait_count_ and Gait_words_ conditions.

Secondary outcome measures included the Timed Up and Go (TUG) test[20], bradykinesia, attention and a serial reaction time task. The TUG was repeated under two dual tasking conditions i) while counting backwards by 3s (TUG_count_) and ii) while generating a list of words starting with a particular letter (TUG_words_). To assess whether dual-tasking improved independent of gait performance, the number of correct responses (numbers or words) in the time taken to complete the TUG was calculated for each dual-tasking condition.

To assess bradykinesia, participants were asked to perform the following sequence 10 times: i) hand closing (squeezing a ball) and opening, ii) elbow flexion, iii) hand closing and opening and iv) elbow extension. The time taken to complete this sequence and the number of errors made was recorded for the right and left sides[15].

Visuomotor speed and procedural learning were examined using a Serial Reaction Time Task[21]. A number from 1–4 was randomly displayed on a computer screen and participants instructed to push a key corresponding to this number as quickly and as accurately as possible. Four blocks of 60 numbers were presented. The first and last blocks contained a random number sequence, whereas the second and third blocks contained the same 12 number sequence repeated 5 times. Reaction time and number of errors were calculated. Visuomotor speed was calculated as the median RT from blocks 1 and 4 (random blocks). Procedural learning was assessed as the reduction in RT of the repeated sequences during blocks 2 and 3 and sequence specific learning as the difference in RT between blocks 4 (random) and 3 (last block with repeated sequences)[22]. Attention was assessed using the Trail-making A and B tests.

To characterize the population, severity of PD was measured using the motor section of Unified Parkinson’s Disease Rating Scale (MDS-UPDRS)[23] and levodopa medication dosage was calculated[24].

### Statistical analysis

An intention to treat analysis was conducted. Data for each variable were compared between groups (active tDCS vs. sham; between subjects factor) and time-points (baseline, post-training and follow-up; within subjects factor) using a two-way, mixed methods analyses of variance (ANOVA). The dual task cost was calculated for each of the gait variables (speed, cadence, step length, double support time) by subtracting values for the gait alone task from the dual tasking activity. These data were analysed using the same ANOVA described above (factors group and time). Data that were not normally distributed were log transformed. Significance was set at p<0.05. Where appropriate, post-hoc analyses were performed using Holm-Sidak tests with correction for multiple comparisons.

## Results

### Feasibility and safety

Participants were enrolled in the trial between October 2013 and January 2014 with follow-up testing complete by April 2014. All participants completed the study with the exception of one individual (active tDCS group) who was unable to attend the follow-up session due to unrelated surgery. One participant experienced an adverse event during training. After 15 minutes of training the individual experienced strong tingling over the site of one electrode and a momentary flash of light in his eyes. The sensations lasted approximately 5 seconds. The participant ceased training that day, but continued on subsequent days with no other events, and no other symptoms. Adherence to training was high, with participants completing 98% (141/144) of all sessions scheduled. Participants reported being within 20% of the instructed attention allocated in the majority of sessions (>56%). The lowest adherence occurred when participants were required to direct the majority (80%) of their attention to improving their gait. Participant characteristics are presented in Table 1. Participants in the active tDCS group had a longer disease duration and poorer UPDRS motor scores at baseline (p<0.05).

**Table 1:** Participant characteristics (mean ± standard deviation).

	tDCS (n = 8)	Sham (n = 8)	P-value
Age (years)	72±4.9	63±11.0	0.054
Gender female:male	0:8	6:2	<0.001
Disease duration (years)	6.9±4.4	4.6±3.9	0.29
Hoehn-Yahr (score:interquartile range [median])	2:2	2:2	1.0
MDS- UPDRS III (score/132)	47.7±7.5	37.7±9.8	0.039
MMSE (score)	29.0±0.76	29.7±0.46	0.031
Levodopa equivalent daily dose (mg)	730±341	523±398	0.142

### Gait

Individuals with PD walked slower and with shorter step length when performing all three dual tasking conditions than in the gait only condition (all p<0.026), indicating dual tasking interfered with gait. A significant improvement was observed for gait speed (effect of time all p<0.035, mean difference ranging 0.06 to 0.20 m/s) and cadence (effect of time all p<0.022, mean difference ranging 2.3 to 7.6 steps/min) under all dual tasking conditions with training and this effect was maintained at follow-up (Table 2). Step length also improved under all dual tasking conditions with training (effect of time all p<0.046, mean difference ranging -0.005 to 0.07 m). This effect was maintained at follow-up for Gait_count_ and Gait_conversation_ but not Gait_words_. Finally, double support time improved under the dual task conditions of Gait_words_ and Gait_conversation_ with training (effect of time all p<0.026, mean difference ranging -0.04 to -0.02 s), and these improvements were maintained at follow-up. There was no improvement in double support time with Gait_count_. The addition of active tDCS did not further enhance dual task training with no difference between the active and sham tDCS groups observed for any gait variable over time (p>0.38). There was no improvement in the number of correct responses over time in either the Gait_count_ (interaction p = 0.22) or Gait_words_ (interaction p = 0.98) conditions for the active or sham tDCS groups (Table 3). There was no difference in the dual task cost for any variables between the active and sham tDCS over time (interaction all p>0.17).

**Table 2:** Gait variables under the gait only and three dual task conditions (count, words and conversation). Data are provided as i) means and standard deviations at each time-point (Pre, Post and Follow-up) for each group, ii) change scores from baseline (Post-Pre, Follow-up-Pre) for each group and iii) 95% confidence intervals between groups for change scores (Post-Pre, Follow-up-Pre).

Variable	Gait only		Count		Words		Conversation	
	tDCS	Sham	tDCS	Sham	tDCS	Sham	tDCS	Sham
Speed (m/s)								
Pre	1.44±0.20	1.35±0.13	1.19±0.22	1.19±0.15	1.18±0.21	1.16±0.16	1.20±0.16	1.22±0.08
Post	1.48±0.17	1.54±0.17	1.25±0.13*	1.39±0.12*	1.28±0.17*	1.35±0.16*	1.34±0.13*	1.40±0.8*
FU	1.48±0.18	1.47±0.15	1.27±0.14*	1.39±0.11*	1.28±0.16*	1.32±0.15*	1.32±0.17*	1.40±0.12*
Post—pre	0.04±0.15	0.19±0.16	0.06±0.15	0.20±0.15	0.10±0.07	0.19±0.17	0.14±0.11	0.18±0.09
FU—pre	0.04±0.12	0.12±0.06	0.08±0.16	0.20±0.10	0.09±0.08	0.16±0.09	0.12±0.17	0.18±0.11
tDCS—Sham [post-pre]	-0.02 to 0.32		-0.02 to 0.30		-0.05 to 0.23		-0.07 to 0.15	
tDCS—Sham [FU-pre]	-0.02 to 0.18		-0.03 to 0.27		-0.03 to 0.17		-0.10 to 0.22	
Cadence (steps/min)								
Pre	111.1±7	116.7±11	97.4±11	109.5±13	98.1±13	110.5±13	104.4±8	113.0±8
Post	110.8±6	120.9±9	99.9±10*	115.7±8*	100.4±11*	116.5±7*	107.3±8*	116.7±8*
FU	108.6±4	119.1±11	99.3±9*	115.9±10*	102.6±8*	114.9±10*	104.7±6*	117.7±9*
Post—pre	-0.34±3.3	4.2±4.2	2.5±7.6	6.2±6.2	2.3±3.7	6.0±6.9	2.9±5.5	3.7±3.3
FU—pre	-2.5±3.5	2.4±2.4	4.5±5.9	6.4±6.1	7.6±4.6	4.4±3.8	2.5±5.4	4.7±3.9
tDCS—Sham [post-pre]	0.49 to 8.59		-3.74 to 11.14		-2.24 to 9.64		-4.06 to 5.66	
tDCS—Sham [FU-pre]	-0.54 to 6.08		-4.82 to 8.62		-7.88 to 1.48		-3.00 to 7.40	
Step length (m)								
Pre	0.76±0.10	0.69±0.08	0.72±0.11	0.64±0.06	0.72±0.11	0.62±0.07	0.68±0.08	0.64±0.07
Post	0.79±0.09	0.75±0.10	0.74±0.09*	0.71±0.07*	0.76±0.12*	0.69±0.09*	0.74±0.08*	0.71±0.07*
FU	0.80±0.11	0.73±0.09	0.76±0.07*	0.70±0.07*	0.73±0.06	0.68±0.09	0.74±0.08*	0.70±0.09*
Post—pre	0.03±0.06	0.06±0.06	0.02±0.04	0.07±0.04	0.04±0.03	0.07±0.05	0.06±0.05	0.07±0.03
FU—pre	0.03±0.04	0.04±0.02	0.03±0.07	0.06±0.02	-0.005±0.08	0.06±0.04	0.06±0.08	0.06±0.04
tDCS—Sham [post-pre]	-0.02 to 0.08		0.01 to 0.09		-0.07 to 0.01		-0.03 to 0.05	
tDCS—Sham [FU-pre]	-0.02 to 0.04		-0.03 to 0.09		0.00 to 0.13		-0.07 to 0.07	
Double support (s)								
Pre	0.24±0.03	0.25±0.03	0.29±0.04	0.28±0.04	0.30±0.05	0.28±0.04	0.29±0.03	0.27±0.03
Post	0.24±0.02	0.22±0.03	0.28±0.02	0.24±0.02	0.28±0.03*	0.24±0.02*	0.26±0.02*	0.23±0.03*
FU	0.23±0.04	0.22±0.03	0.28±0.04	0.24±0.03	0.27±0.04*	0.25±0.03*	0.27±0.03*	0.23±0.03*
Post—pre	0.006±0.02	-0.03±0.02	-0.005±0.03	-0.04±0.03	-0.02±0.02	-0.04±0.03	-0.03±0.06	-0.04±0.06
FU—pre	-0.002±0.02	-0.02±0.01	-0.01±0.04	-0.04±0.03	-0.03±0.02	-0.03±0.02	-0.02±0.02	-0.03±0.02
tDCS—Sham [post-pre]	-0.06 to -0.01		-0.07 to 0.00		-0.05 to 0.01		-0.07 to 0.05	
tDCS—Sham [FU-pre]	-0.04 to 0.00		-0.07 to 0.01		-0.02 to 0.02		-0.03 to 0.01	

* p<0.05 comparison to baseline; FU–follow-up

**Table 3:** Proportion of correct responses (number/time) during each dual tasking condition.

Variable	tDCS	Post-hoc p value	Sham	Post-hoc p value
Gait_count_				
Pre	0.72±0.27	-	0.71±0.18	-
Post	0.71±0.31	N/A	0.55±0.17	N/A
Follow-up	0.66±0.28	N/A	0.72±0.16	N/A
Gait_words_				
Pre	0.63±0.11	-	0.65±0.22	-
Post	0.68±0.10	N/A	0.69±0.15	N/A
Follow-up	0.64±0.10	N/A	0.66±0.17	N/A
TUG_count_				
Pre	0.56±0.23	-	0.68±0.18	-
Post	0.77±0.19	0.017*	0.54±0.12	0.089
Follow-up	0.66±0.20	0.20	0.71±0.25	0.63
TUG_words_				
Pre	0.43±0.13	-	0.60±0.20	-
Post	0.64±0.16	0.015*	0.53±0.08	0.52
Follow-up	0.60±0.14	0.058	0.66±0.14	0.41

N/A = Post-hoc p value not applicable as main effect from ANOVA not significant

* p <0.05 comparison to baseline

### Timed up and go test

**Fig 2:**
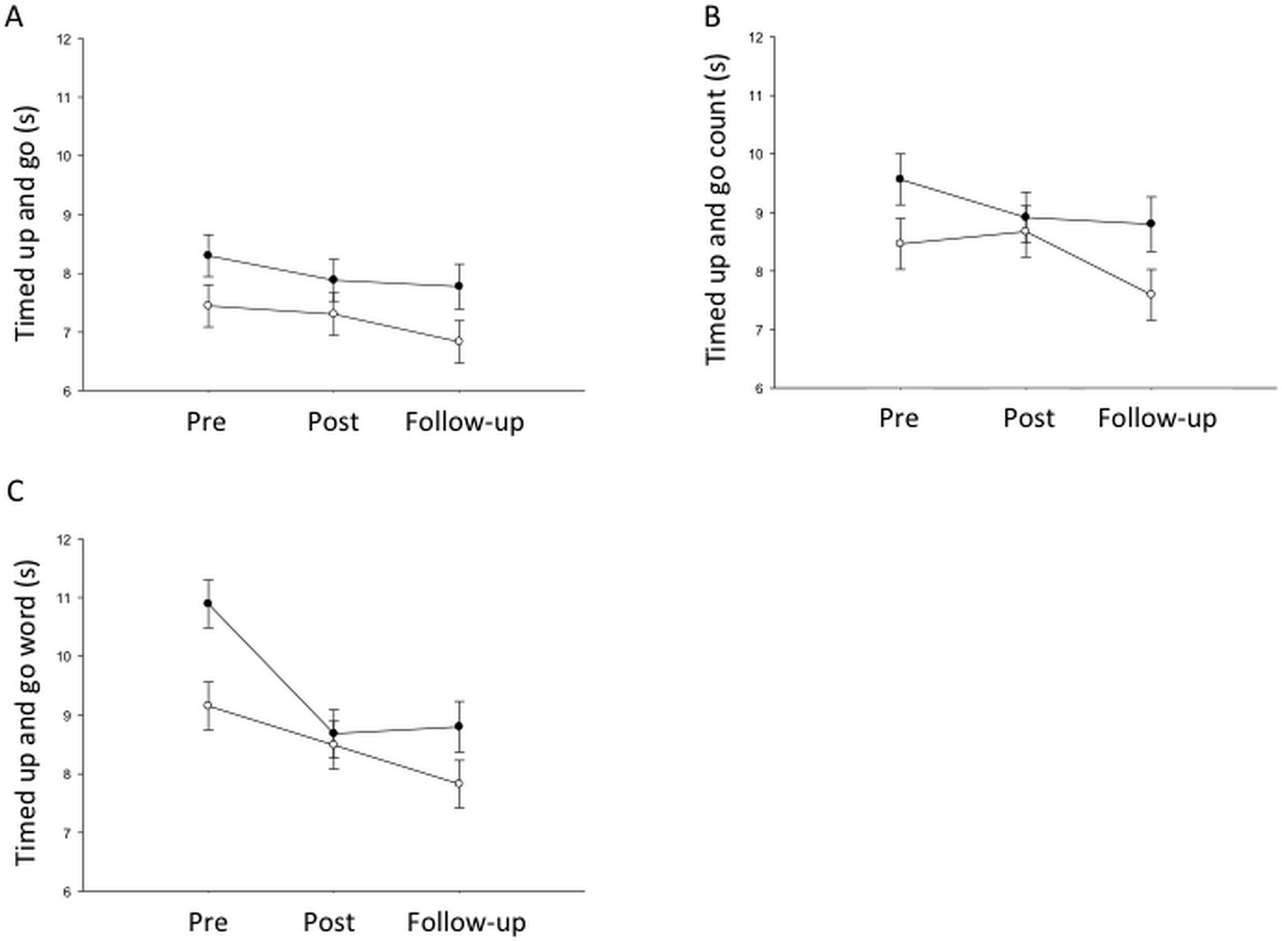
Group data (mean and standard error) for the timed up and go test (TUG) alone (A), the TUG while counting (B) and the TUG while generating word lists (C) in the active tDCS (filled circles) and sham tDCS (open circles) groups. Note the improvement in both groups immediately following training and maintenance of these improvements at follow-up (p<0.05).

The time taken to complete the TUG alone (interaction p = 0.87; time p = 0.31) or in the TUG_count_ condition (interaction p = 0.48; time p = 0.18) was unaltered in either the active or sham tDCS group. Both the active and sham tDCS groups improved the speed with which they completed the TUG_words_ task (interaction p = 0.17; time p<0.001; Fig 2) immediately after training (p = 0.002) and gains were maintained at follow-up (p<0.001). When second task performance was analysed, participants in the active tDCS group improved their correct response rate during both the TUG_count_ (interaction p = 0.003) and TUG_words_ (interaction p = 0.025) dual task conditions immediately following the intervention (Table 3). There was a trend towards maintenance of the improvement in the TUG_words_ task at follow-up (p = 0.058). Similar improvements were not observed in the sham tDCS group.

### Bradykinesia

The time taken to complete the repeated movement sequence decreased in the right arm (interaction p = 0.83; time p<0.001) immediately following training (p = 0.013) and this effect was maintained at follow-up (p = 0.01) in both the active (baseline 37.4±6.5; post 33.1±5.4; follow-up 32.8±4.2) and sham (baseline 35.5±8.9; post 31.2±7.2; follow-up 29.7±5.4) tDCS groups. There was a trend toward a reduction in the time taken to complete the repeated movement sequence in the left arm in both groups over time (interaction p = 0.32; time p = 0.054). The number of movement errors was unchanged in either the right (interaction p = 0.59; time p = 0.65) or left arms (interaction p = 0.13; time p = 0.22).

### Serial reaction time and attention

Reaction time (interaction p = 0.96; time p = 0.12) and error rate (interaction p = 0.081; time p = 0.64) on the serial reaction time task did not change with training in either the sham or active tDCS group. Procedural (interaction p = 0.86; time p = 0.84) and sequence specific learning (interaction p = 0.25; time p = 0.28) were also unaffected by training or type of stimulation (active or sham tDCS). There was no change in the time taken to finish Trail A (interaction p = 0.78; time p = 0.61) or B (interaction p = 0.20; time p = 0.88) in either the active or sham tDCS group immediately following training or at follow-up.

## Discussion

This study is the first to investigate whether anodal tDCS can boost the effect of 3-weeks of dual task gait training with long-term follow-up in Parkinson’s disease. Our data show three weeks of dual task gait training improves aspects of gait performance (speed, cadence, step length, double support time) and the Timed Up and Go test during concurrent language tasks, immediately following training and at 12 weeks follow-up. Improvements in bradykinesia were also observed immediately following and 12 weeks after training. The only parameter that was enhanced by anodal tDCS was the number of correct responses provided while performing the TUG and reproducing word lists or counting. There was no difference between the active and sham tDCS groups for any other measure, indicating that anodal tDCS does not enhance the effect of dual task gait training.

Consistent with previous studies, our data demonstrate worse gait performance in individuals with PD when dual tasking than walking alone[7]. Immediately following training, individuals with PD walked with improved speed, cadence and step length when performing all dual tasks and, with the exception of step length under the Gait_words_ condition, these improvements were maintained at 12 weeks follow-up. In addition, we observed improved double support time for the Gait_words_ and Gait_conversation_ dual tasks and improvements in the TUG_words_ test immediately following, and 12 weeks after, training. These findings extend previous work of dual task training in PD[7, 25, 26] and indicate that three weeks of dual task training is effective at improving gait performance during dual tasking in people with PD. Our data provide further support for the development of gait training programs to improve dual tasking in PD rather than the historical recommendation for individuals with PD to avoid dual tasking activities. The intervention used in this study resulted in improvements in step length and gait speed that are comparable to those seen in previous studies that have explored the effectiveness of cueing-based training strategies in similar populations [27–30]. This is however the first study that has demonstrated that these improvements can be maintained up to 12 weeks after training.

Our data also show an effect of dual task gait training on bradykinesia, with a reduction in the time taken to complete the repeated movement sequence for the right arm immediately following training and at 12 weeks follow-up. A trend toward a similar improvement was present for the left arm. One explanation for these data is that dual task training improved attention and working memory, aspects of cognition that have previously been shown to be associated with bradykinesia in PD[31]. However, we found no change in executive function as measured by the Trail-making tests. These results highlight a need for more detailed exploration of the links between attention, working memory and bradykinesia. Alternatively, the improvement in bradykinesia could represent a motor learning effect as a result of the repeated assessments. We consider this unlikely for two reasons. First, assessments in the current study were spaced 3 (pre/post intervention) and 12 (post intervention/follow-up) weeks apart and second, we observed no effect of training on the serial reaction time test indicating that motor learning (particularly of the upper limb) was not influenced by training.

With the exception of the number of correct responses provided during the dual task TUG conditions, anodal tDCS over M1 did not improve any measure beyond that of sham stimulation when combined with training. Previous literature has shown positive effects of anodal tDCS applied alone on motor function[11], gait[15], and bradykinesia[15] as well as gait speed and balance when combined with physical training[14] in people with PD. Why similar beneficial effects of anodal tDCS were not seen in the present study is unclear.

One explanation is that individuals with PD improved their ability to dual task when walking by decreasing the attention demand of gait (rather than improving motor function per se), allowing them to attend to and practice more challenging added tasks[7]. This hypothesis is consistent with the finding of improved bradykinesia with dual task training. If this is the case, then anodal tDCS may have been more effective if applied to prefrontal brain regions known to be involved in executive function and working memory. Indeed, previous studies have shown improved motor performance, executive function, and working memory when anodal tDCS is applied to the dorsolateral prefrontal cortex in PD [32–34] in addition to reduced cognitive dual-task cost to gait and posture in healthy individuals[35]. Similar improvements in working memory were not observed with anodal tDCS of M1[34].

A recent systematic review concluded that current research using tDCS in people with PD demonstrates an overall positive effect, however the results are not exclusively positive [36], and little is known about the neurophysiological effects of tDCS in people with PD. Furthermore, a recent finding suggests that when applied to the dorsolateral prefrontal cortex, tDCS appears to have a greater effect when applied contralateral to the side of the body with the most severe PD symptoms [32]. In contrast, several studies have reported improved performance with tDCS without unilateral targeting. Gait performance has improved with tDCS applied to the left M1 [15], and Kaski et al [14], reported improved performance when tDCS was applied centrally during physical training, stimulating both hemispheres. In the study presented here, tDCS was always applied to the left M1, and was not targeted at symptoms. When laterality of gait symptoms were investigated in the current study, 10/16 participants did not have a clear unilateral deficit across all gait tasks, making it difficult to choose a side to target.

A previous study has also reported greater tDCS-related improvements in gait and balance in individuals with PD who exhibit more severe motor symptoms[14], which may be due to an increase in M1 activity in people with PD in the later stages of the condition [37]. However, in our study the active tDCS group had greater motor impairment (higher motor UPDRS scores) and a longer disease duration than participants in the sham stimulation group, but still demonstrated no effect. In addition, other studies that have demonstrated an effect of tDCS in PD included participants with similar motor severity scores to the current study^9^, thus the impact of severity and duration of symptoms is unclear and requires investigation.

Finally, it should be noted that other non-invasive brain stimulation interventions such as repetitive transcranial magnetic stimulation (rTMS) have produced variable results on motor function in people with PD. For instance, some studies using high-frequency rTMS of M1 have shown improvements in motor function (UPDRS scores; bradykinesia) and gait velocity in PD[13, 38–40], while others have not[21, 41]. It is not yet clear how inter-individual differences in factors such as disease state, medication or co-morbidities may influence an individual’s response to non-invasive brain stimulation. This inter-individual variability may explain why greater improvements were not observed in the active tDCS condition compared with training alone or why active tDCS did not improve gait alone in the current study. Further research is required to disentangle the factors that impact on response variability in non-invasive brain stimulation trials.

This study should be considered in light of several limitations. First, although powered to detect differences in many variables, our findings are based on a small sample of 16 individuals. Second, all participants were otherwise healthy, living independently in the community and demonstrated relatively good gait performance at baseline, thus it may be important to investigate the impact of tDCS on people with more severe PD. Finally, the stimulation parameters used in the current study were based on previous studies that had reported positive effects[15], albeit with different training tasks. Future investigation is needed to determine optimal stimulation characteristics and dosage when combined with gait training.

## Conclusion

This study provides evidence that 3 weeks of dual task gait training is effective at improving dual task gait performance and bradykinesia, in individuals with PD. These improvements were maintained at 12 weeks follow-up. The addition of tDCS to dual task gait training did not result in greater improvements in gait than training alone, but did positively influenced speed of correct cognitive performance when walking under some conditions. Overall, anodal tDCS applied to M1 may not be an effective adjunct to dual task training in PD.

## Supporting Information

S1 File Study protocol approved by the University of Queensland’s Human Medical Research Ethics committee. (PDF)

S2 File CONSORT statement. (DOC)
