# Supplementary material for: Transcranial Direct Current Stimulation to Enhance Dual-Task Gait Training in Parkinson’s Disease: A Pilot RCT
Source: PLoS One. 2016 Jun 30;11(6):e0158497. doi: 10.1371/journal.pone.0158497 (PMC4928827; doi:10.1371/journal.pone.0158497)
Supplement: S1 File — (PDF) [file pone.0158497.s001.pdf]

CLEARANCE NOº (office use only)

THE UNIVERSITY OF QUEENSLAND

Application Form for Ethical Clearance for Research Involving Human Participants

For review by: **Medical Research Ethics Committee (MREC)**
Behavioural & Social Sciences Ethical Review Committee (BSSERC)

For Staff and Student Research
Refer to last page for website and other information, including mailing address

Please tick boxes:

**MREC**	x	**BSSERC**	

**Full Review**	x	**Expedited Review**	

ALL QUESTIONS MUST BE ANSWERED

- **minimum 12 point font**
- **define any acronyms and abbreviations used**

**Project Title:** Non-invasive brain stimulation for the treatment of gait disturbances in Parkinson's disease

**Principal Investigator:**	Dr Siobhan Schabrun
**Staff Noº/Student Noº:** (cross out if not relevant)	20860918

**Co-Investigator/s:**	A/Prof Sandra Brauer
**Project Co-ordinator (or authorised contact)**	Dr Siobhan Schabrun

**Supervisor/s:** (if applicable)	

**Schools/Departments:**	School of Health and Rehabilitation Science

	Telephone	Fax	Email
**Contact details of Principal Investigator**	54590	51284	s.schabrun@uq.edu.au
**Contact details of Project Co-ordinator or authorised contact**	54590	51284	s.schabrun@uq.edu.au

**Degree Enrolled (if student):**	

**Funding Body:**	
**If Project Funded - What year?**<br>**- Reference no. if available**	

**Project Location:**	School of Health and Rehabilitation Studies (Therapies Building)	**Project Duration:**	4 years

A. Is this submission identical or very similar to a previously approved protocol? **YES/NO** (circle)

If **YES**, please provide clearance noº **and** indicate whether identical or very similar): Similar 2011000613

B. Does this submission hold other ethical clearance? **YES/NO** (circle)

Note: Copies from other **AHEC** registered ethics committees must be attached.

C. Are you applying for Expedited Review? **YES/NO** (circle)

Note: Please see UQ Guidelines page 10 for the conditions necessary to qualify for Expedited Review.

D. Is the project a Clinical Trial (eg, a trial of a drug, device, therapy, intervention, treatment, etc) ? [refer to end of this form dealing with "clinical trials"] **YES/NO** (circle)

If **YES**, please specify: This trial involves a therapeutic intervention in individuals with Parkinson's disease. The intervention consists of a dual task gait training intervention and a non-invasive brain stimulation intervention.

PLEASE ANSWER ALL OF THE FOLLOWING QUESTIONS:

1) Who are the participants or informants?: eg, Children, University students, or other persons.

Note: Details of inclusion/exclusion criteria including approximate **number** (provide justification), age range, and male/female ratios are required.

24 participants will be recruited. All participants will be aged over 18 years (equal male to female ratios) and have a diagnosis of idiopathic PD using the UK Brain Bank criteria. Additional inclusion criteria will comprise: being able to walk 100m independently with or without gait aids; rated stage I-IV on the Hoehn and Yahr disability scale (Hoehn and Yahr 1967); and report reduced step length or slowed gait speed, confirmed by clinical examination. Participants will be excluded if they have metal objects or stimulators in the head that might pose a hazard during tDCS; neurological conditions other than PD; musculoskeletal or cardiopulmonary conditions that affect the ability to safely walk; score < 24 on the Mini-Mental Status Examination (MMSE; Folstein et al 1975) or sensory system pathology affecting walking or communication (e.g. blindness, deafness).

2) Special Groups

The *National Statement* has identified certain groups with specific ethical considerations. Researchers must take special care to protect the interests of these groups if they are in any way involved in the project. Those groups include: **pregnant women and the foetus** (Ch 4.1); **children and young people** (Ch 4.2); **people in dependent or unequal relationships** (Ch 4.3); **people highly dependent on medical care** (Ch 4.4); **people with cognitive impairment, intellectual disability, or mental illness** (Ch 4.5); **people involved in illegal activities** (Ch 4.6); **Aboriginal and Torres Strait Islander peoples** (Ch 4.7); **people in other countries** (Ch 4.8); **other cultural and ethnic groups**.

In preparing your research project and application for ethical clearance, you should investigate thoroughly, through consultation with supervisors, colleagues in your school and other professional groups/organizations, how these special groups may or may not be represented in your research and if participation in this research could have a negative impact on members of any of these groups.

Note: If participation of special groups is a focus of the research, the protocol can not qualify for expedited review (unless other current HREC clearance is held and a copy provided).

2a) Aboriginal and Torres Strait Islanders Group

Specify the level of participation that Indigenous Australians will have in this research (as members of the research team, or as members of the group to be researched):

no participation	some participation possible or likely	focus of the research
x	□	□

Please explain your choice:

Inclusion of Aboriginal and Torres Strait Islanders is not an essential part of this study.

If Indigenous Australians may be involved (2nd or 3rd response box above), what strategies will be used to address their needs and interests? [For guidance with this part of Q2a on indigenous and cultural issues, please refer to the NHMRC and AIATSIS codes of ethics for research with indigenous people. For further advice please contact the UQ Aboriginal and Torres Strait Islander Studies Unit.]

Please specify your strategies:

2b) People in Australia belonging to other cultural or ethnic groups

Are there any ethical considerations that may arise as a result of collection from other cultural or ethnic groups in Australia? [for example, are there any particular customs, practices, or conditions which should be taken into account]: YES/NO

If YES, please provide details:

Have you consulted anyone with knowledge to provide guidance? Who?:

2c) People in overseas countries

Does your project involve data collection in an overseas country?: YES/NO

If YES, what ethical considerations may arise as a result of such data collection, which are different from those arising from data collection in a general Australian context? [for example, are there any particular **local** laws, customs, practices, or conditions which should be taken into account?]:

Have you consulted anyone with knowledge to provide guidance? Who?:

2d) Other Special Groups

Does your project involve any of the other special groups (listed above in the introduction to Q2)?: YES/NO

If YES, please answer the following:

Specify the group/s:
People in Dependent or Unequal Relationships
People with a Cognitive Impairment, an Intellectual Disability, or a Mental Illness

What is the level of their participation:

some participation possible or likely	focus of the research
x	□

What strategies will be used to address their needs and interests?

Please specify your strategies:

People in Dependent or Unequal Relationships

- Health care professionals and their patients or clients – it is anticipated that a proportion of our subjects will be recruited by treating physicians. Subjects will not be coerced into participating in this study, and will be made aware that declining to participate or withdrawing from the study for any reason will not affect their ongoing medical care, or relationship with their health professional.

People with a Cognitive Impairment, an Intellectual Disability, or a Mental Illness

- Cognitive Impairment – Cognitive decline is often associated with Parkinson's disease, therefore it is likely that some of the subjects recruited for this study may have a degree of cognitive impairment. However, this is most common in people with more advanced Parkinson's disease and we are recruiting those with moderate disease severity. Also, participants with severe cognitive impairment will be excluded from the study. We have addressed the issue of some participants possibly having a degree of cognitive impairment by allowing all subjects to have a member of their family or a friend present during explanation of the project. Participants with severe cognitive impairment will be excluded from the study.

3a) Participant recruitment details: Please provide exact details of contact.

Participants will be recruited through Parkinsons Disease Queensland Inc, neurologists who specialise in movement disorders and from lists researchers' hold of people with PD happy to be contacted to be involved in research.

Participants will be targeted within Brisbane and surrounding areas for ease of attendance. An information sheet and consent form detailing the aims, procedures, risks and benefits as well as the inclusion and exclusion criteria will be given to each subject to determine their eligibility and willingness to participate in this study.

3b) Does recruitment include disclosure of personal information (eg, mailing list, names, contact details, etc) from another party or organisation to the researchers? **YES/NO**
If <u>YES</u>, please provide details.
Note: disclosure of personal information from another party or organisation to the researchers, even if merely for the

purpose of seeking initial expression of interest in the project, must be authorised by each individual to whom the information relates (unless it is a completely public database with unrestricted access). Eg, Clinic X must not give to the researchers a mailing list of patients who might be potential participants for the project unless those patients have previously authorised such use and disclosure of their information to non-clinic parties.

4) In EVERY-DAY or LAY LANGUAGE please provide a summary of the project – including aims and benefit: This section MUST be completed in LAY LANGUAGE.

Parkinson's disease (PD) is a progressive neurological disorder characterised by deficits in gait, postural control and learning. Despite intensive investigation, PD remains therapeutically challenging with deficits becoming recalcitrant as the disease progresses. Pharmacological and more recently, surgical approaches provide the basic foundation for treatment in PD. Yet, these approaches are associated with adverse side effects and in the case of surgery, are limited to a small well defined patient population. Physiotherapy training to cue gait (strategy training) has been performed clinically for many years and is now underpinned by increasing evidence (Morris et al 2010). For example, it has been shown that with training, individuals with mild to moderate PD can relearn motor skills such as walking when performing added tasks (dual task gait training; Canning et al 2008; Brauer and Morris 2010). However, functional gains are often not sustained. The addition of non-invasive therapies with the potential to enhance learning and function beyond that of currently available techniques in PD is clearly desirable.

Transcranial direct current stimulation (tDCS) is a powerful non-invasive brain stimulation technique demonstrating early promise as an adjunct therapy in PD. tDCS involves the application of weak direct currents to the cortex via surface scalp electrodes. The application of direct current is painless, safe and cost effective (Poriesz et al 2007). Although the exact mechanism is unclear, tDCS likely targets resting membrane potentials through the manipulation of ion channels and alteration of electrical gradients (Bolognini et al 2009). These shifts in resting membrane potential alter cortical excitability (Nitsche and Paulus 2000) and are thought to underpin improved motor learning and function following direct current applications. Indeed, improvements in motor learning have been demonstrated in healthy individuals (Nitsche et al 2003; Stagg et al 2011) and in those with chronic and subacute stroke, upper limb motor function is improved following tDCS (Lindenberg et al 2010; Kim et al 2010).

Despite the considerable therapeutic promise associated with tDCS, only three studies have examined its effectiveness in PD. These preliminary studies demonstrate improvements in gait, motor function and bradykinesia following one to eight sessions of tDCS over the motor cortex (Benninger et al 2011; Fregni et al 2006; Lomarev et al 1991). Improvements in gait were present immediately following tDCS, while improvements in bradykinesia were maintained three months after therapy (Benninger et al 2011). These studies provide early evidence for the therapeutic potential of tDCS as an adjunct therapy in PD.

It must be noted however, that these studies use a sub-optimal approach to the therapeutic application of tDCS (Bolognini et al 2009). A key feature of tDCS is its ability to modulate cortical excitability. This has led to the exciting suggestion that tDCS may be capable of priming the brain to increase its receptiveness to other therapies. The underlying principle is that motor retraining may be more effective at utilising the neural mechanisms responsible for learning, if motor cortical areas are facilitated prior to training

(Bolognini et al 2009). It is therefore feasible, that priming the brain with tDCS prior to motor retraining may induce greater functional gains than those achieved with motor retraining or tDCS alone. For example, Lindenberg and colleagues (2010) investigated the effect of tDCS combined with standard physical and occupational therapy on motor function in chronic stroke and compared the results with those of standard therapy alone. The authors reported significantly greater improvements in Fugel-Myer (20.7 vs 3.2 %) and Wolf Motor Function Test (19.1 vs 6.0 %) scores when tDCS was used to prime the brain prior to physical and occupational therapy. This suggests that tDCS is most effective when coupled with other therapies. Surprisingly, there have been no studies combining tDCS with other forms of therapy in PD.

One therapy which may be enhanced by the application of tDCS in PD is dual task training. When asked to perform a concurrent task while walking, for example thinking or holding an object, individuals with PD demonstrate reduced gait velocity and step length (Morris et al 1996; Bond and Morris 2000; Galletly and Brauer 2005), increased stride to stride variability (Yogev et al 2007) and more freezing episodes (Spildooren 2010). Recent evidence suggests that individuals with PD can improve their ability to dual task when walking with training (Canning et al 2008; Brauer and Morris 2010). For example, a 20 minute session of dual task training consisting of walking while concurrently performing working memory language (word association and generation) and counting (forward and backward by 2 s and 5 s) resulted in increased step length and gait speed (Brauer and Morris 2010). However, it is unknown whether the addition of tDCS to dual task training in PD will result in greater functional outcomes than dual task training alone.

Thus, we aim to undertake a preliminary study to investigate the effect of tDCS coupled with dual task gait training (speed and step length) in individuals with PD. In addition, we will build on previous research by examining the effect of tDCS on bradykinesia, motor learning and motor function. This study will be the first to combine a promising novel therapy (tDCS), with evidence-based physiotherapy gait training. This line of research has the potential to improve functional outcomes in PD by enhancing currently available therapies. If successful, improved outcomes will translate into greater independence and quality of life for over 4 million individuals living with PD.

5) Give details of the research plan:

Note: The committee needs sufficient information to put into context the ethical considerations listed in later questions.

Note: This section should be completed in <u>LAY LANGUAGE</u> *as much as possible* so that it can be understood and appreciated by all Committee Members, including Lay Members.

Note: For application to the MREC – please keep response to a <u>MAXIMUM</u> of 2 pages.

Experimental Design

A pilot randomised, double-blind, sham-controlled trial with a three month follow up will be undertaken. Participants will be randomised into 2 groups: physiotherapy + tDCS or physiotherapy + sham tDCS.

Participants

Twenty-four people will be recruited from Parkinson's Queensland Inc., neurologists who specialise in movement disorders, and from lists researchers hold of people with PD happy to be contacted to be involved in research. All participants will be aged over 18 years and have a diagnosis of idiopathic PD using the UK Brain Bank criteria. In addition, inclusion criteria will comprise: being able to walk 100m

independently with or without gait aids; rated stage I-IV on the Hoehn and Yahr disability scale (Hoehn and Yahr 1967); and report reduced step length or slowed gait speed, confirmed by clinical examination. Participants will be excluded if they have metal objects or stimulators in the head that might pose a hazard during tDCS; neurological conditions other than PD; musculoskeletal or cardiopulmonary conditions that affect the ability to safely walk; score < 24 on the Mini-Mental Status Examination (MMSE; Folstein et al 1975) or sensory system pathology affecting walking or communication (e.g. blindness, deafness).

Randomisation and blinding

An offsite investigator not involved in recruitment, intervention or data collection will prepare the concealed randomisation using a computer generated random number sequence. Consecutively numbered, randomly ordered opaque envelopes containing group allocation in a 1:1 ratio will be opened consecutively after baseline assessment by the research assistants implementing the intervention. Research assistants who enrol participants, and conduct all assessments will be blinded to group allocation. Participants will not be informed of group allocation. Treating physiotherapists and investigators performing the tDCS intervention will be blind to group allocation.

Intervention

Participants will undergo 8 sessions of training over 3 weeks.

Transcranial direct current stimulation (tDCS):

Direct current will be transferred via saline soaked surface electrodes (35cm2) and a specially developed, battery operated unit (Magstim, UK). tDCS will be applied with the anode positioned over the left primary motor cortex (M1) according to the 10/20 international system for EEG electrode placement and the cathode positioned over the contralateral supra-orbital region (forehead above eyebrows). This montage has been used previously in PD (Fregni et al 2006). A constant current of 2 mA intensity will be applied for 20 minutes immediately preceding the physiotherapy intervention (Benninger et al 2011). For sham stimulation the electrodes will be placed in the same position as for active tDCS. The current will be ramped up over 10 seconds and then ramped down over a further 10 seconds before being switched off. This is a standard sham approach in tDCS studies which ensures that participants feel the initial tingling sensation associated with tDCS (Benninger et al 2011; Lindenberg et al 2011). The tDCS unit will be placed out of sight for both the active and sham interventions.

Physiotherapy: The physiotherapy intervention will be undertaken by a trained physiotherapist on a one-on-one basis, with training commencing at the patient's self-reported optimal 'ON' period, often 1 hour post medication. The dual task gait training program is based on a previous trial (Brauer et al 2011) and will aim to improve gait speed and step length when concurrently performing added cognitive or motor tasks. Participants will undertake repeated practice and cueing of walking aiming to improve step length and speed. Gait tasks will be progressed from simple to more complex tasks, with a variety of added tasks progressively integrated into the training program. These include tasks such as listening, speaking, conversing, generation of simple and complex lists, language, calculation and motor tasks increasing in complexity. Tasks will include those designed to reflect functional everyday activities, such as carrying bags, getting keys out of a pocket, counting money, recalling directions or making a shopping list. Training walking performance will be measured with a trundle wheel. This, and added task performance, will be

recorded in realtime using an iPAD2.

Measures

Measures will be undertaken in the week pre and post intervention, and at three months follow up, approximately one hour after medication, in the 'ON' state. All measures have previously been used by the investigators in people with PD. The primary outcome measure will be comfortable gait speed when walking over 8m and undertaking a concurrent cognitive task, measured post intervention using a GAITrite electronic walkway. Secondary outcome measures include additional parameters of gait (step length), attention (Trail-making A and B tests) (Reitan 1958), visuo-motor speed and procedural learning (serial reaction time task; Benninger et al 2009; Benninger et al 2011), and bradykinesia (Benninger et al 2011). Characteristics of participants will be captured with common clinical tests including: the Hoehn and Yahr scale (Hoehn and Yahr 1967), the Unified Parkinson's Disease Rating Scale (MDS-UPDRS) motor subsection III (Goetz et al 2008), the Freezing of Gait questionnaire (Giladi et al 2009), and the ambulatory self confidence questionnaire (Asano et al 2007), in addition to age, disease duration, medical history, number of falls in previous year, mobility aid use, medication type and dosage.

6) Give details of the ethical considerations attached to the proposed project:

Ethical approval from the MREC has been obtained previously for each of the procedures listed in this proposal. Detailed ethical considerations for each technique are given below:

Transcranial direct current stimulation: tDCS is a non-invasive, painless and safe technique which is used worldwide. The use of tDCS in healthy subjects and across a range of pathological conditions has not resulted in any significant adverse effects[8]. tDCS produces a mild tingling or itching sensation over the scalp when the machine is first turned on. This sensation subsides within the first couple of minutes. Mild symptoms of fatigue (35 %), mild discomfort (15 %), headache (5 %) and nausea lasting not more than 2 hours (3 %) have been reported following tDCS[8]. Subjects will be fully briefed on each of these symptoms and be given the opportunity to withdraw at any time. The presence of these symptoms will be monitored verbally by the investigator and tDCS terminated if the subject becomes uncomfortable. Subjects who have a history of frequent or severe headaches/migraines will be excluded from participation.

Dual task gait training: Dual task gait training will be performed one-on-one with a trained physiotherapist. Participants will use their standard walking aid (if any) at all times and be instructed to walk at a safe, comfortable pace. As participants who are unable to walk 100 m independently (with or without gait aids) will be excluded from this study, any risk of falling is no greater than that encountered in everyday life.

Measurements of gait speed and step length: As participants who are unable to walk 100 m independently (with or without gait aids) will be excluded from this study, any risk of falling during these measurements (walking over 10 m) is no greater than that encountered in everyday life. Participants will be able to use their normal gait aid and will be instructed to walk at a comfortable, safe pace.

Serial reaction time task and tests of bradykinesia: There are no risks associated with these measurements.

Questionnaires: There are no risks associated with any of the questionnaires utilised in this study.

7a) How will informed consent be obtained from participants or informants?

Participants will be provided with an information statement and consent form. The investigator will also verbally outline the procedures to be used in the study and any risks involved. Any questions related to these forms will be answered. Consent forms will be signed prior to commencement of the study. .
Those subjects who are unable to give their written consent due to physical impairments will be asked to give verbal consent, and written consent will be obtained from a significant other.

7b) "Gatekeeper" Approvals

A "gatekeeper" or "permission-giver" is a person authorised to write a Letter of Authority and Recognition from an organisation of any type involved with the research, which gives permission to the researcher for access to the population **under the "gatekeeper's" or "permission-giver's" authority**.

[For example, if you wish to conduct research in schools and the participants are the school teachers, then gatekeeper approval will need obtained from the relevant education authority (eg, Education Queensland) and the School Principals before you may approach those school teachers in recruitment.

For example, if you wish to access staff from a private organisation, then similarly, gatekeeper approval will usually be required from senior personnel or an appropriate manager who is able to grant such access to approach that organisation's staff in recruitment.]

1. Are gatekeeper approval/s required for the research?: **YES/NO**

2. If **YES**, who are the gatekeeper/s and how will their approvals be sought and obtained? (if gatekeeper approval/s have already been obtained, then please attach copy)

8) Provide details of procedures for establishing confidentiality and protecting privacy of participants or informants:

Each participant will be allocated an identification code in order to protect his or her identity. Any data collected will then be recorded with respect to the identification code. Personal information collected will be stored in a secure database with access only by investigators involved in the research. The information obtained from this experiment will be treated with confidentiality. Publications resulting from this study will reveal the information in a manner which does not identify the individuals involved. Participants will be informed of both the potential risks and confidentiality issues within the information sheet and consent form. At all times, the confidentiality of subjects, the personal information and the recorded data will be maintained at the standards in accordance to the National Health and Medical Research Council's guidelines.

9) Researchers must ensure that all data, particularly data containing personal information (ie, information that can identify the person), are secure both at the point of storage and during transit. Researchers must be aware of relevant legislation and guidelines governing privacy:- *Information Privacy Act* (Qld) 2009, *Privacy Act* (Cth) 1988, and Guidelines under S95 and S95A of the *Privacy Act* (Cth).

9a) Where will data be stored (eg, UQ office of researcher), and what measures will be taken to ensure security of data (eg, locked filing cabinets, computer hard-drive protected by password/encryption/de-identification of data, etc)?

All hardcopies of data will be kept in a locked filing cabinet in the office of the chief investigator for the duration of the study when not in use. Computer data will be de-identified and stored in a personal drive of a university computer, with a backup burnt to CD which will be kept with the hard copy data in a locked cabinet.

9b) Will data be stored on, or taken to, premises other than secure UQ premises (eg, researcher's home)?: YES/NO (NO circled)

If YES, then what measures will be taken to ensure security of data at these premises?

9c) What measures will be taken to ensure security of data during transit? (eg, if data is on hard-drive – protection by password/encryption/de-identification of data, etc).

9d) Will persons other than staff of the research team have access to the data?: YES/NO (NO circled)

If YES, then please specify these persons, state why these persons have access, and what provisions are in place to ensure the confidentiality of data by these persons.

10) In what form will the data be <u>collected</u>:

Note: Tick the most appropriate box:		
**(i) Identified** x	**(ii) Potentially Identifiable** ☐	**(iii) De-Identified** ☐ (ie, not able to be re-identified)

**11) In what form will the data be <u>stored and/or accessed</u>:** Note: Tick the most appropriate box:		
**(i) Identified** ☐	**(ii) Potentially Identifiable** ☐	**(iii) De-Identified** x (ie, not able to be re-identified)

**12) Give details of how feedback will be available to participants or informants:**
If requested, a report of performance on clinical and laboratory measures will be made available to participants as soon as possible following testing.

13) Does the project involve any of the following possibilities? Answer YES or NO. If YES, give details.

a) The trial or use of any medicine, drug, or other substance

1. Answer YES or NO. If YES, provide details: No
2. Does this project require the submission of a Clinical Trial Notification/Clinical Trial Exemption (CTN/CTX) Form to the Therapeutic Goods Administration (TGA)? [Refer to the TGA website for further information]:

b) The trial of any device

1. Answer YES or NO. If YES, provide details: Yes. Transcranial direct current stimulation will be trialled in participants with Parkinson's Disease.
2. Does this project require the submission of a Clinical Trial Notification/Clinical Trial Exemption (CTN/CTX) Form to the Therapeutic Goods Administration (TGA)? [Refer to the TGA website for further information]: No

c) The trial of any intervention, therapy, or treatment (whether medical, behavioural, physical, or other)

Yes. Transcranial direct current stimulation will be trialled in conjunction with dual task gait training (physiotherapy intervention) in participants with Parkinson's Disease.

d) Any invasive procedures (eg, blood sampling)

No

e) Any diagnostic scans carried-out for the purposes of the project (including, *but not limited to*: MRI, NMR, CT/CAT, X-Rays, etc).

1. If YES, please list.

No
2. Does your project involve the use of MRI? YES/NO<br><br>NOTE: If using MRI at a hospital site (i.e. a facility with emergency services available on site during testing), you MUST have at least one staff who has current CPR certification and must have undertaken an emergency evacuation drill at least once a year.<br><br>If using MRI at non-hospital sites, (e.g. UQ St Lucia Campus), you MUST have 2 staff who both have current CPR certification and they must have undertaken an emergency evacuation drill at least once a year.<br><br>Does your project fulfil these mandatory conditions? YES/NO<br><br>If NO, outline reasons for submitting your application without these conditions in place.
3. Does your project involve exposure to ionising radiation? YES/NO<br><br>NOTE: If YES, the protocol MUST comply with the Queensland *Radiation Safety Act (1999)* and *Radiation Safety Regulation (2010)*. The legislation requires compliance with the Australian Radiation Protection and Nuclear Safety Agency's *Code of Practice for the Exposure of Humans to Ionising Radiation for research Purposes (ARPANSA 2005)* (http://www.arpansa.gov.au/pubs/rps/rps8.pdf) and you MUST consult with the University Radiation Protection Adviser before submission.<br><br>Does your project meet the guidelines of the Code of Practice? YES/NO<br><br>Has the project been reviewed by the University Radiation Protection Adviser before ethics submission? YES/NO

f) The possibility of physical stress/distress, or discomfort

1. to the participants:<br>This project involves some risks, inconveniences and discomforts associated with tDCS of the motor cortex. These have been comprehensively outlined above and in the information sheet attached. The project has been designed to address our research questions in such a manner that the participants are exposed to the minimum possible degree of risk, inconvenience and discomfort.<br><br>2. to the researchers/data collectors:<br>There is no potential for physical stress/distress or discomfort to the researchers/data collectors.

g) The possibility of psychological/mental stress/distress, or discomfort

1. to the participants:<br>There is no potential for psychological stress/distress or discomfort to the participants.<br><br>2. to the researchers/data collectors:<br>There is no potential for psychological stress/distress or discomfort to the researchers/data collectors.

h) Deception of/or withholding information from, participant at **ANY** stage of the project

No

i) Access, by the investigators, to data held by a Commonwealth Department or Agency (Please also specify the number of records to be accessed)

No

j) Access, by the investigators, to data held by other bodies or people (Please also specify the number of records to be accessed)

No

k) Access to data (eg, medical records), by other bodies or people not the investigators.

No

l) Use of questionnaires, interviews, or focus groups with questions or topics which are sensitive, have potential to cause distress, or may reveal illegal activity

No

14) Please Indicate What You Think Is The Level Of Risk For Prospective Participants Against The Scale Below: *Tick the most appropriate box. (Refer to the UQ Guidelines)*

	**Extreme Risk**
	**High Risk**
	**Some Risk**
x	**Minimal Risk**
	**No Foreseeable Added Risk Above the Risks of Everyday Living**

15) Please provide details to assist the committee as to why you indicated the level of risk to prospective participants or informants in the question above (Question 14):

tDCS is a painless, non-invasive procedure which carries minimal risks, inconveniences or discomforts. Published safety guidelines will be adhered to all times[8]. The presence of mild symptoms of fatigue, headache and nausea will be explained to each subject and monitored verbally throughout the experiment. The subject will have the opportunity to withdraw at any time. All investigators on this project are fully qualified physiotherapists with current first aid and CPR certificates. All have been thoroughly briefed in on UQ emergency procedures.

All walking tasks associated with dual task training and measurement of gait function will be performed at a comfortable, safe pace with the participant’s normal walking aid. Participants who are unable to walk independently over 100 m will be excluded from this study.

16) How has the possibility of withdrawal from the project been addressed?:

Note: Ensure that details and effects of withdrawal without prejudice AT ANY TIME have been considered and explained. Refer to the NHMRC’s *National Statement* section 2.2.19 – 2.2.20.

Participants will be informed that they are free to withdraw from the study at any time, without stating a reason, without affecting their relationship with the researchers.

17) Please note that this section must be completed for funded research or the application will not be processed.

**17 a) Is this project receiving financial support to conduct the research?**	**YES/NO** (circle) — YES circled
**17 b) If Yes, from what source(s)?**	
Parkinson's Disease QLD Inc	
**17 c) Who will be administering the budget?**	
SM Schabrun and SG Brauer (The University of Queensland)	
**17 d) Please provide details of the budget distribution. (Or attach a copy of the budget statement.)**	
Please see attached.	
**17 e) Provide details of any other "in kind" support for the project or direct or indirect payment to any investigator:**	
N/A	
**17 f) Please provide details of participant reimbursement for their involvement in the Project, if any:** **Note:** This could be cash payment, food vouchers, free services, or movie passes, etc.	
Travel to and from assessment and treatment sessions are often a barrier for people with PD being involved in research. We have previously found a mixture of providing both taxi transport and parking options will suit potential volunteers.	

**18) In undertaking this research do any "conflict of interest" issues arise?** **If YES,** please provide details. **Note:** Conflict of Interest may arise, for example, because a researcher, or someone close to the researcher, stands to benefit financially from the research or the carrying out of the project or because inconsistent or incompatible obligations exist. Refer to section 5.4 of the NHMRC's *National Statement*:
No

**19) Is the project a multi-centre or site project?** **If YES**, provide the name of the principal ethics committee. Please provide copies of any conditions or requirements placed by other AHEC registered Human Ethics Committees: **Note:** The Principal Ethics Committee is the Institutional Ethics Committee where the budget is to be administered.
No

**20a) Some projects may involve permits from National Parks & Wildlife in relation to collection of data and**

**Native Title issues. How have you addressed this issue?:** (Refer to the UQ Guidelines)
N/A
**20b) Does the project require biosafety clearance?** **YES/NO** (circle)

ATTACHMENTS:

1) Participant Consent Form **Yes/No**

Note: for examples of what should be included in a consent form, please consult page 12 of the UQ Guidelines for Ethical Review of Research Involving Humans. Also refer to "Checklist" below.

2) Participant Information Sheet **Yes/No**

Note: for External Use - forms should be released on letterhead and contain University Ethical Paragraph.

Refer to UQ Guidelines and Ethics website, and "Checklist" below.

3) Questionnaire (if applicable) **Yes/No**

4) Indemnity Agreement (primarily for clinical trials and contract work) **Yes/No**

5) CTN/CTX [Clinical Trial Notification/Clinical Trial Exemption] Form **Yes/No**
(primarily for clinical trials)

6) Gatekeepers or Permission-Givers **Yes/No**

Note: A "gatekeeper" or "permission-giver" is a person authorised to write a letter of Authority and Recognition from an organisation of any type involved with the research, which gives permission to the researcher for access to the population under the "gatekeeper's" or "permission-giver's" authority.

7) Bibliographic References **Yes/No**

8) Other - please specify Budget statement attached

DECLARATION

We/I, the undersigned researcher(s) have read the University of Queensland's Guidelines for Ethical Review of Research Involving Humans and the NHMRC's *National Statement on Ethical Conduct in Human Research*, and agree to abide by them in the conduct of this research. It is understood that this includes the reporting and monitoring roles associated with the approval by the University of Queensland.

Signature of Principal Investigator:

Date: 23 / 01 /2012

Signature of Supervisor (if applicable)**:** N/A

Date: / /

An original plus 15 copies should be submitted to the:

Ethics Officer
Research & Innovation Division
Cumbrae-Stewart Building (72)
THE UNIVERSITY OF QUEENSLAND QLD 4072

Ph: (07) 3365 3924
Fax: (07) 3365 4455

Email: humanethics@research.uq.edu.au

ADDITIONAL INFORMATION

Application information, including the UQ Guidelines, can be found on our website:
http://www.uq.edu.au/research/rid/human-ethics

The NHMRC's *National Statement* can be found on the following website:
http://www.nhmrc.gov.au/publications/synopses/e72syn.htm

Information concerning clinical trials and the CTN/CTX schemes can be found on the TGA website:
http://www.tga.gov.au/index.htm

Information regarding biosafety can be found on the following website:
http://www.uq.edu.au/ohs/biosafety-at-uq

Aboriginal and Torres Strait Islander Studies Unit website: http://www.uq.edu.au/atsis/ (which includes links to sites including the Australian Institute of Aboriginal and Torres Strait Islander Studies Unit under Cool Sites). Enquiries to the Aboriginal and Torres Strait Islander Studies Unit can be made on: 3365 6714 (ext 56714).

Full Review of applications may take a minimum of eight weeks from the time of submission. Expedited Review and Amendments may take a minimum of three weeks.

NHMRC: National Health and Medical Research Council
AHEC: Australian Human Ethics Committee
HREC: Human Research Ethics Committee and, for the purposes of this application, means an AHEC registered committee

Applications to MREC
Please note that medical research includes epidemiological research (Privacy Act 1988).

Audits

Please note that the Committee reserves the right to visit the research site and view materials at any time, and to conduct a full audit of the project.

Last Update 08/11/2011

Submission of Research Protocols for Human Ethical Clearance
APPLICATION CHECKLIST

This checklist is supplied for use as an additional means of ensuring all aspects of the proposed study have been considered and adequately detailed before submission to a reviewing Committee. A copy must be attached to the original application form for the reviewing Committee to support your submission.

Project Title:
Principal Investigator:

Participant Information Sheet (PIS)

	YES	NO	IF NO, WHY?
1. Version for each participant group *(if applicable)*		X	n/a
2. On letter-headed paper *(if applicable)*	x		
3. Full title of project	x		
4. Lay title of project *(if applicable)*		x	n/a titles are the same
5. Names, positions & affiliations of all investigators	x		
6. Clear purpose of study	x		
7. Non-technical language - Appropriate lay language and length for PIS	x		
8. Details of participation/ procedures	x		
9. Duration of participation	x		
10. Location for participation	x		
11. Risks outlined *(% explanation needed?)*	x		
12 Benefits to participants	x		
13 What support if something goes wrong	x		
14. Freedom to withdraw without penalty	x		
15. Assurance of confidentiality	x		
16. Access to results	x		
17. Debriefing	x		
18. Reimbursement to participants	x		
19. Need for Witnesses?		x	n/a
20. Contact details for further questions	x		
21. Ethical clearance statement	x		

Participant Consent Form (PCF)

	YES	NO	IF NO, WHY?
1. Version for each participant group *(if applicable)*		X	n/a
2. Full title of project	X		
3. Lay title of project *(if applicable)*		X	n/a
4. Names, positions & affiliations of all investigators	x		
5. Provision of space for full name of participant	x		
6. Written declaration of informed consent, eg, "I have read/"I understand…"	x		
7. Freedom to withdraw without penalty	x		
8. Assurance of confidentiality	x		
9. No benefit for participation	x		
10. Provision for signature of participant	x		
11. Provision for signature of witness and date (if appropriate)	x		
12 Provision for signature of guardian, relationship to participant and date (if appropriate)	x		

THE UNIVERSITY OF QUEENSLAND
AUSTRALIA

School of Health and Rehabilitation Sciences
Division of Physiotherapy

The University of Queensland
Brisbane Qld 4072 Australia
Telephone (07) 3365 2008
International +61 7 3365 2275
Facsimile (07) 3365 2775

PARTICIPANT INFORMATION SHEET

TITLE: Non-invasive brain stimulation for the treatment of gait disturbances in Parkinson's disease

INVESTIGATORS: **Dr Siobhan Schabrun**
Division of Physiotherapy, University of Queensland
A/Prof Sandra Brauer
Division of Physiotherapy, University of Queensland

1. Subject selection and purpose of study

You are invited to participate in a study of transcranial direct current stimulation and dual task gait training for the treatment of gait disturbances in Parkinson's disease. You will be excluded from participation in the study if you have implanted metallic or stimulating devices, are unable to walk 100 m independently (with or without a gait aid), other neurological, cardiopulmonary, sensory or musculoskeletal conditions that affect your ability to walk safely or score < 24 on the Mini-Mental Status examination. We hope to learn whether transcranial direct current stimulation is useful as an adjunct treatment to dual task gait training in Parkinson's disease.

As a participant in these studies, you will be asked to attend 11 sessions with each session lasting between one and three hours. Assessments will be conducted at the Human Neuroscience Unit in Room 522 at the School of Health and Rehabilitation Sciences, The University of Queensland, St Lucia Campus. You will be reimbursed for your parking or travel costs.

2. Description of study and risks

If you agree to participate in this study the following procedures will be performed.

- ☐ Transcranial direct current stimulation (tDCS) is a non-invasive, painless and safe technique used to alter the electrical activity of various brain regions positioned under a stimulating electrode. The technique involves two electrodes; one applied over the region of your brain controlling movement and the other above your eye. A low level electrical current is passed between the two electrodes to induce a change in electrical activity. You may feel a mild tingling or itching sensation when the machine is first turned on. However, this should quickly subside. Minimal risks are associated with this

technique. A small number of individuals may experience fatigue, mild discomfort, headache or nausea following tDCS. These symptoms should disappear within 2 hours. If you experience any discomfort and wish to stop the stimulation please let the investigator know.

- ☐ Dual task gait training will be undertaken by a trained physiotherapist on a one-on-one basis, with training commencing at your self-reported optimal ‘ON’ period (often 1 hour post medication). The dual task gait training program will aim to improve gait speed and step length when concurrently performing added thinking or movement tasks. You will undertake repeated practice and cueing of walking aiming to improve step length and speed. Walking tasks will be progressed from simple to more complex tasks, with a variety of added tasks progressively integrated into the training program. These include tasks such as listening, speaking, conversing, generation of simple and complex lists, language, calculation and motor tasks increasing in complexity. Tasks will include those designed to reflect functional everyday activities, such as carrying bags, getting keys out of a pocket, counting money, recalling directions or making a shopping list. There are no risks associated with this training program.

- ☐ Gait measurements (measurements of walking). You will be asked to walk at a comfortable speed (using your usual gait aid if you have one) over an 8 m electronic walkway while undertaking a concurrent thinking task (e.g. number calculations). We will measure your walking speed and step length. There are no risks associated with these measurements.

- ☐ Reaction time task. You will be instructed to press a key on a keyboard corresponding to the numbers 1-4 as quickly and as accurately as you can. You will be asked to complete 7 blocks of 120 keyboard presses. There are no risks associated with this task.

- ☐ You will be asked to make a number of hand and arm movements (squeezing a ball, opening and closing your hand, bending and straightening your elbow) as quickly as you can 10 times. There are no risks associated with this task.

- ☐ You will be asked to fill in a number of common questionnaires including the Freezing of gait questionnaire and the ambulatory self confidence questionnaire to monitor your walking progress during the study. There are no risks associated with these questionnaires and you will not be asked to provide any information that might be considered sensitive.

The studies are designed to address our aims in such a manner that you are exposed to the minimum possible degree of risk, inconvenience and discomfort. However, you will have the opportunity to withdraw from these procedures at any time should you wish to do so, without penalty or affecting the ongoing management of your condition in any way. Because this is a research project and not a treatment program, there may not be any direct benefit to you from your involvement. However the findings may help us to develop more effective therapy techniques for people with musculoskeletal pain.

Feedback on individual assessment results will be provided on request and a summary of the overall outcomes of the study will be available at the completion of the research project.

Should you have any questions regarding the nature of the research, please feel free to contact Dr Siobhan Schabrun (3365 4590) who will be happy to provide you with more information.

Your privacy while participating in this study will be maintained at all times. Any publications will not allow identification of any individual, and, our data will be identified by a numerical code, not by your name. Files will be kept in a locked filing cabinet in the Human Neuroscience Unit in the Division of Physiotherapy at the University of Queensland.

This study has been cleared by one of the human ethics committees of the University of Queensland in accordance with the National Health and Medical Research Council's guidelines. You are of course, free to discuss your participation in this study with project staff (contactable on 3365 4590). If you would like to speak to an officer of the University not involved in the study, you may contact the Ethics Officer on 3365 3924.

Thank you for your interest in this research project.

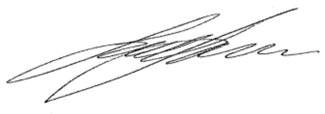

Dr Siobhan Schabrun

THE UNIVERSITY OF QUEENSLAND
AUSTRALIA

School of Health and Rehabilitation Sciences
Division of Physiotherapy

The University of Queensland
Brisbane Qld 4072 Australia
Telephone (07) 3365 2008
International +61 7 3365 2275
Facsimile (07) 3365 2775

CONSENT FORM

TITLE: Non-invasive brain stimulation for the treatment of gait disturbances in Parkinson's disease

INVESTIGATORS: **Dr Siobhan Schabrun**
Division of Physiotherapy, University of Queensland
A/Prof Sandra Brauer
Division of Physiotherapy, University of Queensland

1. I, ____(PLEASE PRINT) hereby consent to take part in the research project titled: ***Non-invasive brain stimulation for the treatment of gait disturbances in Parkinson's disease***

2. I acknowledge that I have read the information sheet provided, and that I have had the project, so far as it affects me, fully explained to my satisfaction by the investigators. I freely consent to my participation in the project.

3. The details of the procedure proposed has also been explained to me, including the anticipated length of time it will take, the frequency with which the tests will be performed, and an indication of any discomfort or possible risks which may be expected. I understand that the tests to be taken are as follows:

- ***Transcranial direct current stimulation***
- ***Dual task gait training***
- ***Gait measurement***
- ***Reaction time task***
- ***Measures of hand movement speed***
- ***Questionnaires***

4. Although I understand that the purpose of this research is to improve the quality of medical care, it has also been explained that this is a research project and not a treatment program, and my involvement may not be of any direct benefit to me.

5. I am informed that the results of any tests involving me will not be published so as to reveal my identity and that my privacy will be maintained at all times.

6. I understand that I am free to withdraw from the project at any stage without penalty and that this will not affect in any way the ongoing management of my condition.

Signed: __________________________ Name: _________________________ Date: _______________
Signed: ___________________________ Name: _________________________ Date: ______________
(witness) (Print)

GENERAL HEALTH QUESTIONNAIRE

1. Name: ....

2. Date of Birth: ....

3. Height: ....cm

4. Weight: ....kg

5. Are you left or right handed? (Please circle) LEFT/RIGHT

6. Have you ever experienced a seizure? Please circle YES/NO

7. Have you ever experienced any faintness, light-headedness, blackouts? YES/NO

8. Have you ever had a stroke? YES/NO

9. Have you ever had a head injury (or neurosurgery)? YES/NO

10. Do you suffer from frequent or severe headaches? YES/NO

11. Have you ever had any illness that caused brain injury? YES/NO

12. Are you taking any medications at the moment? (except PD meds)? YES/NO
If yes, please specify medication type and condition being treated:

- ....
- ....
- ....
- ....
- ....

13. Have you had any operations or surgical procedures of any kind performed in the last 15 years? YES/NO
If yes, please list them:

- ....
- ....
- ....
- ....
- ....

14. Do you have diabetes? YES/NO
If yes, how is it controlled?..

15. Do you have any heart conditions? YES/NO
If yes, please specify:..

16. Do you have any implanted devices such as cardiac pacemakers, medical pumps, or intracardiac lines? YES/NO
If Yes, please specify:..

17. Do you have any other systemic or inflammatory diseases or illnesses? YES/NO
If yes, please specify:...

18. Do you have any infections or infectious disease? YES/NO

19. Do you have any allergies? YES/NO

20. Have you had back surgery? YES/NO

21. Have you had abdominal surgery? YES/NO

22. Do you have or have you had: (Please tick)

Gout ❑
Epilepsy ❑
Arthritis ❑
Muscular Pain ❑
Hernia ❑
Asthma ❑
Glandular Fever ❑
Rheumatic Fever ❑
Stomach Ulcers ❑
Liver condition ❑
Kidney condition ❑
Heart Murmur ❑
High Blood Pressure ❑
Chest pain/palpitations ❑
High Cholesterol ❑

Respiratory condition ❑ Specify:..
Neurological condition ❑ Specify:..

23. Do you smoke? YES/NO
If yes, how long have you smoked?..
How many per day? ....

24. Have you ever experienced Urinary Incontinence? YES/NO
If yes, how long ago? ....

25. Do you experience urinary urgency (a feeling of needing to urinate often without much flow of urine)? YES/NO

26. Do you experience consistent constipation? YES/NO

27. How would you rate your level of everyday physical activity? Please tick.
□ *Minimal* – that required to complete activities of daily living. (e.g. Washing clothes, walking to work/mailbox, cooking/cleaning).
□ *Moderate* – activities such as sports (e.g. exercise classes, golf, social tennis etc.), gardening.
□ *High* – activities that exhaust you (e.g. vigorous cycling, competitive sport etc)

28. Have you had a fall in the last 12 months? YES/NO
If yes, how many? ....
How and where did they occur? ....
....
....

SF-36 SUB-SCALE

29. During the past 4 weeks, to what extent has your physical health or Emotional problems interfered with normal social activities with family, friends, neighbours, or groups? (Please tick)

□ Not at all □ Slightly □ Moderately □ Quite a bit □ Extremely

30. How much bodily pain have you had during the past 4 weeks? (Please tick)

□ No bodily pain □ Very mild □ Mild □ Moderate □ Severe □ Very Severe

For Women Only

31. Are you post menopausal? YES/NO

32. Are you receiving hormone replacement therapy? YES/NO

I certify that the above information is correct to the best of my knowledge. I have read and understand the entire conents of this form and I have had the opportunity to ask questions regarding the information on this form.

Participant's name: ____

Participant's signature: ______________________ **Date:** ___________

Transcranial Magnetic Stimulation Questionnaire (Wassermann 1998)

1. Have you ever experienced transcranial magnetic stimulation (TMS)? YES/NO

If yes, did you have any adverse reactions? YES/NO

2. Have you ever experienced a seizure or fainting spells? YES/NO

3. Have been medically diagnosed with epilepsy? YES/NO

4. Does anyone in your family have epilepsy? YES/NO

5. Have you ever had an EEG (to measure brain activity)? YES/NO

If yes, please give details.

6. Have you ever had a stroke? YES/NO

7. Have you ever had a head injury (including neurosurgery)? YES/NO

8. Have you ever lost consciousness? YES/NO

9. Do you suffer from frequent or severe headaches? YES/NO

10. Have you ever had any other brain-related condition? YES/NO

11. Have you ever had any illness that caused brain injury? YES/NO

12. Are you taking any medications at present? YES/NO

If yes, please give details.
13. Have you had any operations or surgical procedures of any kind performed in the

last 15 years? YES/NO
Please list them.
-
-
-
-
-
14. If you are a female, are you currently pregnant? YES/NO

15. Have you ever experienced any faintness, light-headedness, blackouts? YES/NO

16. Do you have diabetes? YES/NO

If yes, how is it controlled?

17. Do you have any heart conditions? YES/NO

18. Do you have any metal in your head (outside of the mouth), such as shrapnel, surgical clips, or fragments from welding or metalworks? YES/NO

19. Do you have any implanted devices such as cardiac pacemakers, medical pumps, or intracardiac lines? YES/NO

20. Do you wear a hearing aid? YES/NO

21. Do you have any other systemic or inflammatory diseases or illnesses? YES/NO

22. Have you had fine-wire electrode inserted anywhere in the body before? YES/NO

23. Have you ever fainted before during blood testing or any other procedures involving needle insertion? YES/NO

24. Do you have any bleeding or clotting disorders? YES/NO

I certify that the above information is correct to the best of my knowledge. I have read and understand the entire contents of this form and I have had the opportunity to ask questions regarding the information on this form.

Participant’s name:

Participant’s signature:

Date

References

Asano M et al. Development and psychometric properties of the ambulatory self-confidence questionnaire. Gerontology 2007, 53:373-381.

Benninger DH et al. Transcranial direct current stimulation for the treatment of Parkinson’s disease. J Neurol Neurosurg Psychiatry 2010, 81: 1105-11.

Benninger DH et al. Safety study of 50 Hz repetitive transcranial magnetic stimulation in patients with Parkinson’s disease. Clin Neurophysiol 2009, 120: 809-15.

Bolognini N et al. Using non-invasive brain stimulation to augment motor training-induced plasticity. J Neuroengin Rehab 2009, 6:8.

Bond JM and Morris M. Goal directed secondary motor tasks: their effects on gait in subjects with Parkinsonian disease. Arch Phys Med Rehab 2000, 81: 110-6.

Brauer SG and Morris M. Can people with Parkinson’s disease improve dual tasking when walking? Gait & Posture 2010, 31:229-33.

Brauer SG et al. Single and dual task gait training in people with Parkinson;s disease: a protocol for a randomised controlled trial. BMC Neurol 2011, 11: 90.

Canning CG et al. Multiple task walking training in people with mild to moderate Parkinson’s disease: a pilot study. Clin Rehabil 2008, 22: 226-33.

Carter JH et al. Living with a person who has Parkinson's disease: the spouse's perspective by stage of disease. Parkinson's Study Group. Mov Disord 1998, 13:20-28.

Dorsey ER et al. Projected number of people with Parkinson disease in the most populous nations, 2005 through 2030. Neurology 2007, 68:384-386.

Folstein MFet al. "Mini-Mental State" A practical method for grading the cognitive state of patients for the clinician. J Psychiatric Research 1975,12:189 - 98.

Fregni F et al. Noninvasive cortical stimulation with transcranial direct current stimulation in Parkinson’s disease. Mov Disord 2006, 21: 1693-702.

Galletly R and Brauer SG. Does the type of concurrent task affect preferred and cued gait in people with Parkinson’s Disease? Aust J Physiother 2005, 51: 175-80.

Giladi N et al. Validation of the freezing of gait questionnaire in patients with Parkinson's disease. Mov Disord 2009, 24:655-661.

Goetz CG et al. Movement Disorder Society-sponsored revision of the Unified Parkinson's Disease Rating Scale (MDS-UPDRS): scale presentation and clinimetric testing results. Mov Disord 2008, 23:2129-2170.

Hoehn MM and Yahr MD. Parkinsonism: onset, progression and mortality. Neurology 1967, 17:427-442.

Kim DY et al. Effect of transcranial direct current stimulation on motor recovery in patients with subacute stroke. Am J Phys Med and Rehab 2010, 89:879-86.

Lindenberg et al. Bihemispheric brain stimulation facilitates motor recovery in chronic stroke patients. Neurol 2011, 75: 2176-84.

Lomarev MP et al. Method of Parkinsonism treatment. Intervention SU 1630836 (Russian), Bulletin Isobreteni 8 (1991).

Morris M. Locomotor training in people with Parkinson's disease. Phys Ther 2006, 86:1426-35.

Morris ME et al. Striding out with Parkinson disease: evidence-based physical therapy for gait disorders. Phys Ther 2010, 90:280-288.

Nitsche MA et al. Facilitation of implicit motor learning by weak transcranial direct current stimulation of the primary motor cortex in the human. J Cogn Neurosci 2003, 15: 619-26.

Nitsche MA and Paulus W. Excitability changes induced in the human motor cortex by weak transcranial direct current stimulation. J Physiol 2000, 527: 633-9.

Poreisz C et al. Safety aspects of transcranial direct current stimulation concerning healthy subjects and patients. Brain Res Bull 2007, 72:108-114.

Reitan R: Validity of the Trail Making Test as an indicator of organic brain damage. Percept Mot Skills 1958, 8:271-276.

Schrag et al. How valid is the clinical diagnosis of Parkinson's disease in the community? J Neurol Neurosurg Psychiatry 2002, 73:529-534.

Spildooren et al. Freezing of gait in Parkinson's disease: the impact of dual-tasking and turning. Mov Disord 2010, 25: 2563-70.

Stagg CJ.Polarity and timing-dependent effects of transcranial direct current stimulation in explicit motor learning. Neuropsychologia 2011, 49:800-804.

Yogev G et al. Dual tasking, gait rhythmicity and Parkinson's disease: which aspects of gait are attention demanding? Eur J Neurosci 2005: 22:1248-56

Detailed Budget *(please list budget items in priority order, with 'A' denoting essential to the success of the project)*

Research assistant (HEW level 5, 02)		
- blinded assessor	A	$6,483
- tDCS intervener	A	$4,322
- Physiotherapy intervener	A	$8,644
**Equipment** (items > $1,000)		
**Consumables**		
**Travel**		
Parking and transport for participants		
-Parking	A	$1,500
-Taxi costs	A	$13,860
**Other**		
Ipad2 (32 GB, Wifi + 3G)	B	$839
2x advertisment (Parkinson's Qld newsletter)	B	$200
Total funding required		$35,848
Note - this includes a $6000 cash contribution provided by researchers		
**Total Funding Requested**		**$29, 848**

12. Budget Justification

Personnel - Research assistance

A blinded assessor is required to ensure rigor of the experimental design. This person would be a physiotherapist experienced in recruitment, data collection and entry of this type of information in people with PD. The intervention will also require considerable hours that is beyond that of the investigators (over 380 hrs). Research assistants experienced in these interventions in similar trials will be employed.

Blinded assessor, recruitment, data entry: 24 people x 3 sessions x 2hrs x $45.02/hr (UQ rates - HEW 5(02) are $38.48/hr + 17% oncosts = $45.02/hr) = $6,483
tDCS intervener: 24 people x 8 sessions x 0.5hr x $45.02/hr = $4,322
physio intervener: 24 people x 8 sessions x 1hr x $45.02/hr = $8,644

Travel - Parking & transport

Travel to and from assessment and treatment sessions are often a barrier to people with PD being involved in research. We have previously found a mixture of providing both taxi transport and parking options will suit potential volunteers. The parking rates are for a multistorey carpark at the Princess Alexandra Hospital where assessment and training would take place.

10 people x 11 sessions x $15 (PAH parking) = $1500
14 people x 11 sessions x $90 (Taxi rates are currently $2.10 booking fee, $3.20 flag fall and $1.97 per km. An average 20 km one-way trip is likely to cost $44.70.) = $13,860

Other

Data collection

An iPAD2 (32GB, Wi-Fi + 3G) will be used by the physiotherapist to record training performance as they are walking around with the patient. Data will be entered into a database and held in a secure repository. Previously, paper files have been used which has resulted in many hours of data entry. Cost $839.

Recruitment

Recruitment will include contacting people with PD who have indicated that they are happy to be contacted to be involved in research. In addition, two, quarter page advertisements ($100 each) will be placed in the Parkinson's Queensland Inc newsletter. This has been a fruitful recruitment method previously.

Advertisement with Parkinson's Queensland Inc: 2 advertisements at $100 each = $200

Total: $35,848

<u>UQ “CLINICAL TRIAL” INSURANCE</u>

What Constitutes a Clinical Trial (for insurance purposes)?

If the answer is 'YES' to **either** 1 or 2 below, the study or research is a clinical trial for the purposes of the University’s insurance coverage:

1. Is the study or research to test:
   - a drug, **or**
   - a surgical procedure or device; **or**
   - a therapeutic procedure or device; **or**
   - a preventative procedure or device; **or**
   - a diagnostic procedure or device;

   where the nature of the study or research is such that it requires the investigator or an assistant to be a registered medical practitioner or other registered qualified health service provider?

2. Does the study or research require any:

   - penetration of the skin (other than taking of blood samples); **or**
   - biopsy or any taking of or extraction of tissue samples; **or**
   - penetration of the bodily orifices (other than ears or mouth); **or**
   - insertion of diagnostic or other device within the bodily orifices (other than ears or mouth).

   to be undertaken be a registered medical practitioner or other registered qualified health service provider?

However, please note that if the study or research:

- involves evaluating outcomes of established health care management or treatment relating to the condition or illness from which the participants are suffering; **or**
- only involves the participants completing questionnaires or interviews;

then the study/research is not a clinical trial for the purpose of insurance coverage.

An **<u>INSURANCE CHECKLIST</u>** on the next page is provided to assist you in determining whether your project is a ‘clinical trial”.

ALL applicants must complete this checklist.

Further information can be found at the following UQ Insurance Website:
http://www.fbs.uq.edu.au/index.html?page=78431#What%20Constitutes%20a%20CTN%20Trial

INSURANCE CHECKLIST

(**ALL** applicant to complete)

A. Does the nature of the study or research require that the investigator or an assistant thereto must be a registered medical practitioner or other registered qualified health service provider? **Yes x No** ☐

(i) If Yes to A, Is the study or research to test

- a drug, **or**
- a surgical procedure or device; **or** **Yes x No** ☐
- a therapeutic procedure or device; **or**
- a preventative procedure or device; **or**
- a diagnostic procedure or device;

If **Yes** to A and (i) above, then it is a Clinical Trial.

B. Does the study or research require any "invasive procedure" (please note the definition below) to be undertaken by a registered medical practitioner or other registered qualified health service provider?

Yes ☐ **No x**

For the purpose of this question, "invasive procedure" shall mean any procedure involving

- penetration of the skin (other than taking of blood samples); **or**
- biopsy or any taking of or extraction of tissue samples; **or**
- penetration of the bodily orifices (other than ears or mouth); **or**
- insertion of diagnostic or other device within the bodily orifices (other than ears or mouth).

If **Yes** to B above, then it is a Clinical Trial

C. Does the study or research require or involve the submission of a Clinical Trial Notification/Clinical Trial Exemption (CTN/CTX) Form to the Therapeutic Goods Administration (TGA)? [Refer to the TGA website for further information]

Yes ☐ **No x**

If **Yes** to C above, then it is a Clinical Trial

If your project is a "clinical trial" in accordance with the definition above, then please go on to complete the **<u>INSURANCE ANNEXURE</u>** on the next page.

The Ethics Office will detach and forward the completed annexure to the UQ Insurance Office so that it can arrange for insurance on your behalf. The Insurance Office will carry-out any negotiation on behalf of UQ and will bear the cost of the policy.

Researchers for CTN/CTX clinical trials (ie, answered "YES" to C in the checklist above) must, in addition, confirm with the UQ Insurance Office (Ph 3365 3075; email insurance@bs.uq.edu.au) that clinical trial insurance arrangements are in place before commencement of the project, <u>IRRESPECTIVE</u> of UQ Ethics Clearance.

Finance and Business Services Division

THE UNIVERSITY OF QUEENSLAND

Clinical Trial Insurance Notification Form

(to be completed for each Clinical trial and submitted with Ethics application)

**UQ Ethics Number:**	

**Ethics Approval Granted By:**	**UQ** ☐ **Approved** x **Pending**<br>**Other Institution** ☐ **(State if approved or pending, and name of other institution)**……………………………………………………<br>………………………………………………………………….....

**Principal Investigators Name:**	**Siobhan Schabrun**

**UQ Employee [E], Conjoint [C], Adjunct [A], Other (specify):**	**E**

**UQ School/Unit:**	**SHRS**

**Trial Title:**	Non-invasive brain stimulation for the treatment of gait disturbances in Parkinson's disease

**Brief Description:**	Transcranial direct current stimulation (tDCS) is a cutting edge non-invasive brain stimulation technique with the potential to improve function and learning in neurological conditions. However, evidence in PD is limited to three small pilot studies, all of which use sub-optimal tDCS protocols. Here we aim to undertake a preliminary trial to investigate the effect of tDCS coupled with dual task training on gait disturbances in PD. In addition we will examine the effect of tDCS on bradykinesia, motor function and learning. This novel and innovative research has the potential to improve the function and quality of life for over 4 million individuals living with PD.

**Issues required to be specifically notified due to exceptions to UQ's coverage under its Unimutual protection:**	Does the trial/study involve a permanently invasive procedure (e.g. an implant)?	☐ Yes X No
	Does the trial/study involve a pregnant or breast feeding subject?	☐ Yes X No
	Does the trial/study involve a minor?	☐ Yes X No
	Does the trial/study involve the use of any medicine or device not entered in the Australian Register of Therapeutic Goods, including any new formulation of an existing product or any new route of administration?	X Yes ☐ No

	Does the trial/study involve the use of any marketed medicine or device used beyond the conditions of its Therapeutic Goods Administration marketing approval, including new indications extending the use of the product to a new patient group and the extension of doses or duration of treatments outside the approved range?	☐ Yes XNo
	Does the trial/study involve the discontinuation of any existing treatment or medication?	☐ Yes X No
	Does the trial/study involve Implanon being administered?	☐ Yes X No
	Is there a significant risk that the trial/study will result in any person contracting HIV or AIDS?	☐ Yes X No
	Is there a significant risk that the trial/study will result in the transmission of any other communicable or contagious disease or virus?	☐ Yes XNo
	Is there a risk that the trial/study will result in damage to or change in any subject's DNA?	☐ Yes X No
	Is all or part of the trial/study being conducted in the USA or Canada?	☐ Yes X No
	Does any agreement applicable to the trial/study state that the laws of the USA or Canada apply?	☐ Yes X No

Please also note that Unimutual protection may not apply to a trial/study in circumstances involving:

- dishonest, fraudulent, criminal or malicious acts or omissions;
- the performance of services by any individual under the influence of intoxicants, narcotics or other drugs affecting neuro cognitive competence;
- health care incidents where a health care professional's capacity is in question (under the *Medical Indemnity (Prudential Supervision and Product Standards) Act 2003* (Cth));
- the provision of health care by an unregistered individual;
- sexual harassment, sexual misconduct or unlawful discrimination of any type;
- failure of the drug, device or procedure to which the trial/study relates to perform its intended purpose or function; and
- any trial/study undertaken without the approval of all relevant ethics committees.

If any of these circumstances arise at any time before or during the trial/study then you must notify the Director of the Research and Innovation Division immediately.

**Sponsor of Trial:**	**N/A**

**Indemnity provided by sponsor:** (list any exclusions if any):	**N/A**

**Granting body for non-sponsored Trials:**	**Parkinson's disease QLD Inc**

**Estimated target participant numbers per annum** **(Divide no. for full trial period by no. of years for trial)**	**24**

**Estimated target participant numbers for full trial period:**	**24**

**Location (s) of Trial:**	**UQ St Lucia**

**Invasive nature of trial:**<br>eg taking blood samples, tissue sampling, surgical procedures, ingestion of any substance, application of creams, ointments etc:	**None**

**Start date of Trial:**	**Feb 2012**

**Estimated period of Trial:**	**18 months**

**Type of Clinical Trial:**<br>**General** ☐(i.e. Non CTN/CTX)<br>**CTN** ☐ **Phase:** ....<br>**CTX** ☐ **Phase:**	**If CTN/CTX, (once UQ Ethics has been approved, send <u>email</u> to <u>insurance@bs.uq.edu.au</u> with following attachments:**<br>- **Questionnaire (refer "On-line forms" at <u>www.fbs.uq.edu.au</u>)**<br>- **Patient Information Sheet**<br>- **Patient Consent Form**<br>- **If overseas sites involved, copy of full protocol (overseas sites are <u>NOT</u> automatically covered)**

**Name of drug(s) being used:**	**None**

**Dosage of drug(s):**	**N/A**

Signed by: Date: 23/01/2012

(Principal Investigator)

<u>Contact details:</u>

Name of Contact Person: Siobhan Schabrun

Telephone number: (07) 3365 4590

Email address: s.schabrun@uq.edu.au
